# Anatomy of the ligaments of the posterolateral corner of the knee: A narrative literature review

**DOI:** 10.1002/jeo2.70562

**Published:** 2025-11-28

**Authors:** Xavier Angelats, Anna Carrera, R. Shane Tubbs, Joe Iwanaga, Marc Franco, Keishiro Kikuchi, Francisco Reina

**Affiliations:** ^1^ Medical Sciences Department, Clinical Anatomy, Embryology and Neuroscience Research Group (NEOMA) University of Girona School of Medicine Girona Spain; ^2^ Department of Neurosurgery Tulane University School of Medicine New Orleans Los Angeles USA; ^3^ Department of Neurology Tulane University School of Medicine New Orleans Los Angeles USA; ^4^ Department of Structural & Cellular Biology Tulane University School of Medicine New Orleans Los Angeles USA; ^5^ Department of Surgery Tulane University School of Medicine New Orleans Los Angeles USA; ^6^ Department of Anatomical Sciences St. George's University St. George's Grenada; ^7^ Department of Neurosurgery and Ochsner Neuroscience Institute Ochsner Health System New Orleans Los Angeles USA; ^8^ University of Queensland Brisbane Australia; ^9^ Department of Orthopaedic Surgery 12 de Octubre Universitary Hospital Madrid Spain; ^10^ Department of Orthopaedic Surgery Kurume University Kurume Fukuoka Japan

**Keywords:** clinical anatomy, knee anatomy, knee ligaments, posterolateral corner, posterolateral knee surgery

## Abstract

The posterolateral region of the knee or so‐called posterolateral corner of the knee or posterolateral angle is an anatomical entity that remains relatively unknown. A clinical consensus relative to the evaluation, diagnosis and treatment of posterolateral corner injuries has recently been published. However, there are still knowledge gaps as to the precise anatomy and variability of all the structures that are part of this anatomical region. There is also paucity relative to their precise histological characteristics and their function. In addition, the nomenclature used in the literature for the posterolateral corner structures is sometimes confusing. A narrative review of the research focused on the posterolateral corner of the knee over the last hundred years was conducted. The main objective of this work was to synthesize and to bring order to the existing knowledge of the main ligamentous and tendinous components of the posterolateral corner of the knee. Twenty fresh‐frozen human knees from a body donor programme were dissected to obtain anatomical images from each one of the posterolateral corner structures. Currently, there is no consensus regarding the anatomical descriptions of some of the posterolateral corner structures. Their nomenclature is still confusing, and the anatomical descriptions are not sufficiently complete. However, the images taken of each of the posterolateral corner structures show their main morphological characteristics. This work will help to develop a common framework and a consensus on the anatomy of these structures. Currently, the lack of consensus relative to the anatomy of the posterolateral region of the knee makes it difficult to resolve issues around injuries to them, their diagnosis, and the treatment of these structures.

**Level of Evidence:**

N/A.

## Graphical abstract

Review of knowledge about anatomy and terminology of the posterolateral corner of the knee, highlighting the need for consensus to improve clinical understanding and treatment of related injuries. Figure legend: ligaments of the posterolateral corner of the knee.

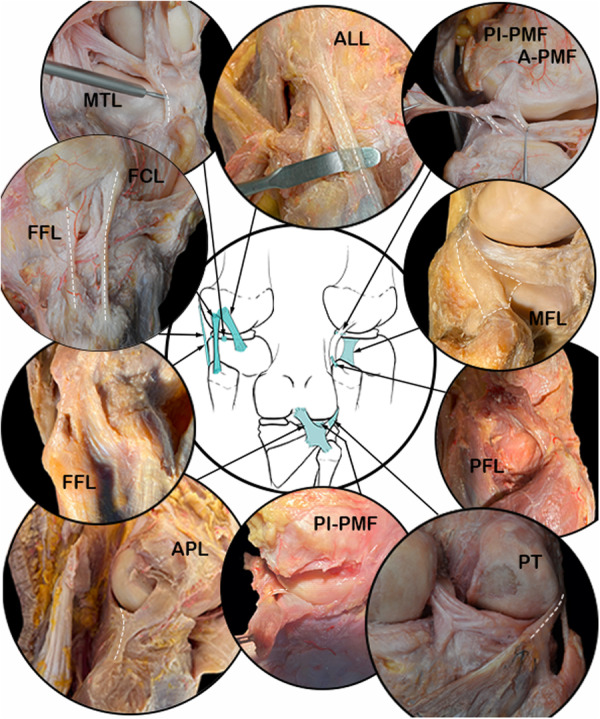

**Abbreviations**
ALL: Anterolateral ligament
APL: Arcuate popliteal ligament
A‐PMF: anterior popliteomeniscal fascicle
FCL: fibular collateral ligament
FFL: Fabellofibular ligament
FIPAT: Federative International Programme for Anatomical Terminology
ITT: iliotibial tract
LMTL: lateral meniscotibial ligament
MFL: meniscofibular ligament
MFmL: meniscofemoral ligaments
MRI: magnetic resonance imaging
MTL: Meniscotibial ligaments
MTPFC: menisco‐tibio‐popliteus‐fibular complex
PFL: Popliteofibular ligament
PI‐PMF: postero‐inferior popliteomeniscal fascicle
PLC: posterolateral corner
PMTL: posterior meniscotibial ligament
PS‐PMF: postero‐superior popliteomeniscal fascicle
PT: popliteus tendon
TA2: Terminologia Anatomica (2nd edition)
US: ultrasound

## INTRODUCTION

The posterolateral corner of the knee (PLC) shows a complex and highly variable anatomy. It is mainly due to evolutionary changes in the anatomical relationships between the structures that form it. The PLC is the most poorly understood anatomical region of the knee [12]. The anatomical complexity of the structures that comprise this region, along with the confusing terminology used to refer to them, has sparked the interest of researchers, anatomists and orthopaedic surgeons in recent years [3, 50, 80, 101]. Although most of the anatomical structures that comprise the PLC have been described in the literature (some quite recently [58]), there is no consensus about their accurate anatomical description regarding their bony landmarks and attachments, their anatomical constitution and structure, and the relationships among them. The fact that there is currently no decision‐making algorithm for the diagnosis and treatment of injuries affecting the PLC nor a surgical technique with expert consensus as to the technique of choice for acute or chronic instability of the PLC shows that this anatomical region remains relatively unfamiliar. However, a novel consensus statement on the evaluation, diagnosis, and treatment of injuries to the PLC from a clinical point of view has been recently published [52].

The PLC has been classically defined as the *dark side of the knee* [14]. This incomplete knowledge is the primary factor that contributes to the poor results currently achieved in treating these knee injuries. This lack of understanding of the PLC often leads to incorrect diagnoses in diagnostic imaging techniques [27, 59, 66], as well as the failure of conservative treatments in undiagnosed high‐grade injuries [25, 49]. That lack also contributes to getting inferior surgical results when the method does not restore the natural anatomy and biomechanics of these structures [51]. At present, there is still no standardized algorithm for the diagnosis and treatment of PLC lesions [14], but the most reliable technique seems to be the anatomical reconstruction of the Fibular collateral ligament (FCL), the Popliteus tendon (PT), and the Popliteo‐fibular ligament [52].

Different works have determined that PLC injuries may cause previously unexplained knee instabilities [31, 46, 48, 62, 104]. The PLC structures are the primary stabilizers in terms of forces in varus, external rotation and posterior translation [67]. Understanding the precise anatomy and biomechanical properties of the PLC structures is important for clinical examination and anatomical reconstruction techniques [86]. The most common mechanisms in PLC injuries are those involving hyperextension (with or without a direct impact), direct anteromedial trauma, and varus forces. Injuries to the PLC structures often occur in the context of other ligament lesions, which result in a multi‐ligament knee injury [39]. Most PLC injuries occur conjointly, either with anterior or posterior cruciate ligament injuries [39, 66]. The failure to diagnose and treat posterolateral knee instability in these patients is the most common identifiable cause of anterior cruciate ligament reconstruction failure after surgery [70].

In total knee arthroplasty, preserving the anatomy of the PLC is essential to avoid posterior dislocations after surgery. This is especially the case when a valgus deformity correction is done in which the posterolateral structures are released to achieve satisfactory realignment [11].

Ultrasound (US) and Magnetic resonance imaging (MRI) are feasible alternatives for a practical approach to the PLC [74]. Both can provide valuable information regarding the anatomy of and injuries to this compartment even though most radiology specialists do not know the pattern of lesions to them [9, 12, 14, 56, 78, 87, 100, 105, 107]. Moreover, the inconsistent terminology used to describe PLC structures increases the confusion surrounding this anatomical entity [12, 74].

In the present work, a narrative review of the research done over the last century with a focus on the posterolateral corner of the knee has been conducted. The work summarizes the main features of the ligamentous and tendinous components that the literature considers a part of the posterolateral corner of the knee. Through the dissection of 20 fresh‐frozen adult human knees from a body donor programme, a catalogue of images that illustrate the main characteristics of these structures has been elaborated to help the reader to understand each of them. This has made it possible to create a common framework and consensus on the anatomy of these structures.

## THE ANATOMY OF THE PLC OF THE KNEE REVISITED

In the last 50 years, there have been many descriptions of the PLC. From a functional point of view, some authors classify the anatomical structures of the PLC into dynamic and static stabilizers [12]. Others talk about primary and secondary stabilizers [13]. From a topographical point of view, most authors classify the PLC structures in three layers, the superficial, intermediate and deep [8, 12, 98, 107]. To better understand this region, the focus of this review has been placed on the ligamentous components, myotendinous structures and their topographical organization.

### Ligamentous components of the PLC

The ligaments that have been considered part of the PLC by the different authors are the FCL, the Fabellofibular ligament (FFL), the Anterolateral ligament (ALL), the Arcuate popliteal ligament (APL), the Popliteofibular ligament (PFL), the meniscofibular ligament (MFL), the Meniscotibial ligaments (MTL), the Meniscofemoral ligaments (MFmL) and the Posterior cruciate ligament [8, 12, 26, 35, 39, 58, 67, 68, 98, 107].

#### Fibular collateral ligament

The FCL is one of the most extensively studied structures of the PLC. It has been done from an embryological point of view [35, 94], from the structural point of view [45] as well as from the comparative anatomy point of view [48, 66].

**Figure 1:**
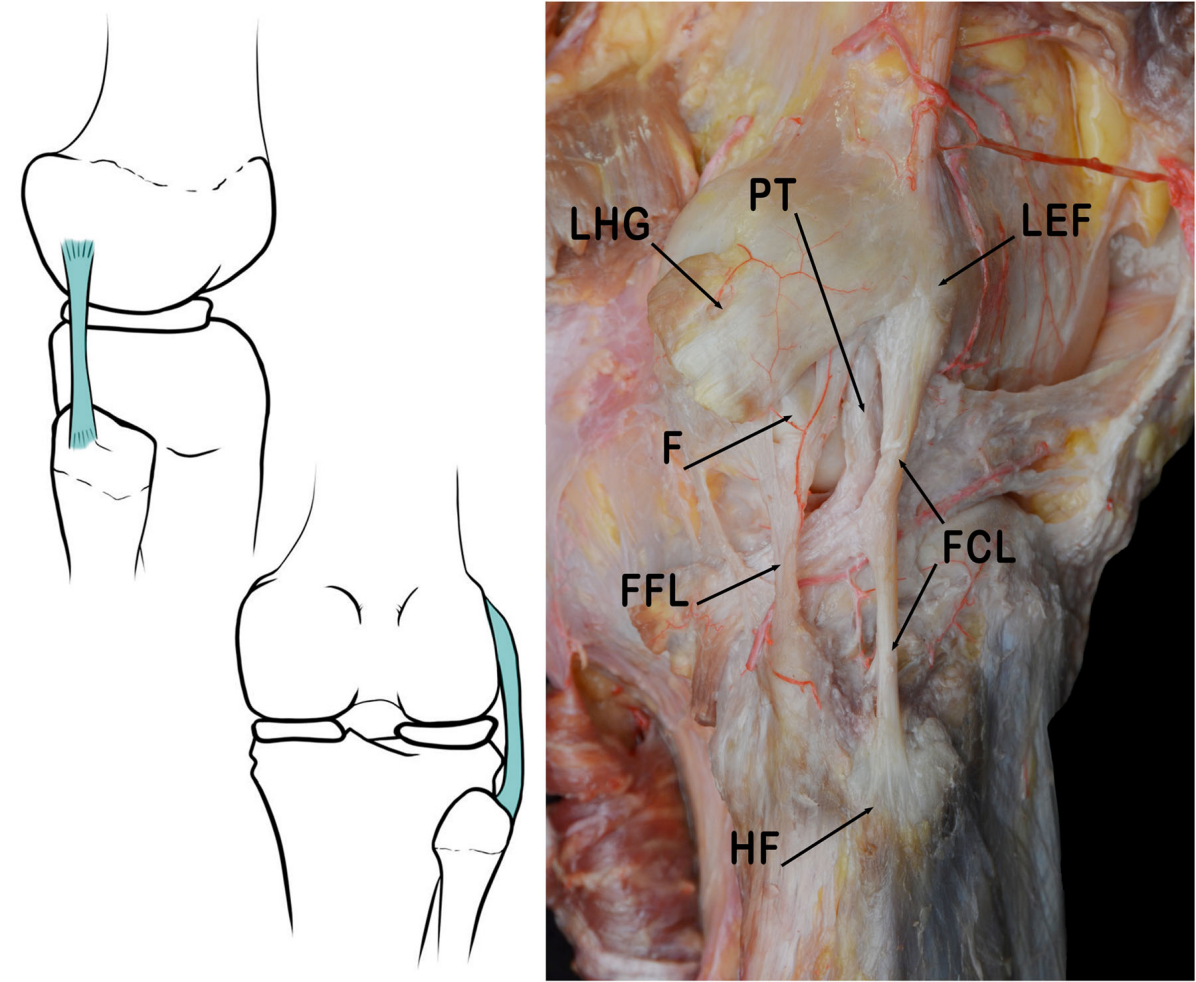
Schematic drawing and anatomical dissection image corresponding to a lateral view of the knee joint to show the fibular collateral ligament (FCL). F, fabella; FFL, Fabellofibular ligament; HF, head of fibula; LEF, lateral epicondyle of femur; LHG, lateral head of gastrocnemius; PT, popliteus tendon.

The FCL connects the femur to the fibula. Its attachment on to the femur is on the proximal and posterior aspect of the lateral femoral condyle. The primary attachment resides in a slight bony depression posterior to the lateral epicondyle. Some fibres extend proximally and anteriorly over the lateral epicondyle in a fan‐like manner. The average distance between the femoral attachment of the FCL and the femoral attachment of the PT is about 1.8 cm [48]. It extends about 7 cm caudally across the femorotibial joint and inserts onto the fibular head [45, 94]. The attachment of the FCL covers approximately 38% of the total width of the fibular head (anterior to posterior from the anterior edge of the fibular head). However, other authors describe and underline the inconsistency in the insertion of this ligament on the fibular head or distal insertion [48] (Figure 1).

This ligament is considered the primary stabilizer of varus forces from 0° to 30° in the knee joint. The FCL, together with the PT and the External gastrocnemius tendon, is considered the primary posterolateral stabilizer in the PLC of the knee by most authors [39, 48]. This ligament, together with the PT and PFL are considered primary structures in the PLC. There is a 100% consensus that their reconstruction and the repair of the secondary restraints can yield satisfactory outcomes [52].

The second edition of the *Terminologia Anatomica* (TA2) defines the accepted denomination that should be used for this ligament, the FCL. Nonetheless, it also accepts Lateral collateral ligament as a synonym [22]. Then again, that this ligament has been given many and varied names during its academic study must be considered. It was initially described as the degeneration of the tendon of the Fibularis (peroneus) longus muscle [29, 93]. In classical terminology, it was named the Long external lateral ligament, and later, it was called the Lateral knee ligament. Afterward, it was also called other names: lateral fibular ligament, lateral collateral fibular ligament, or external collateral ligament [48, 53].

#### Fabellofibular ligament

**Figure 2:**
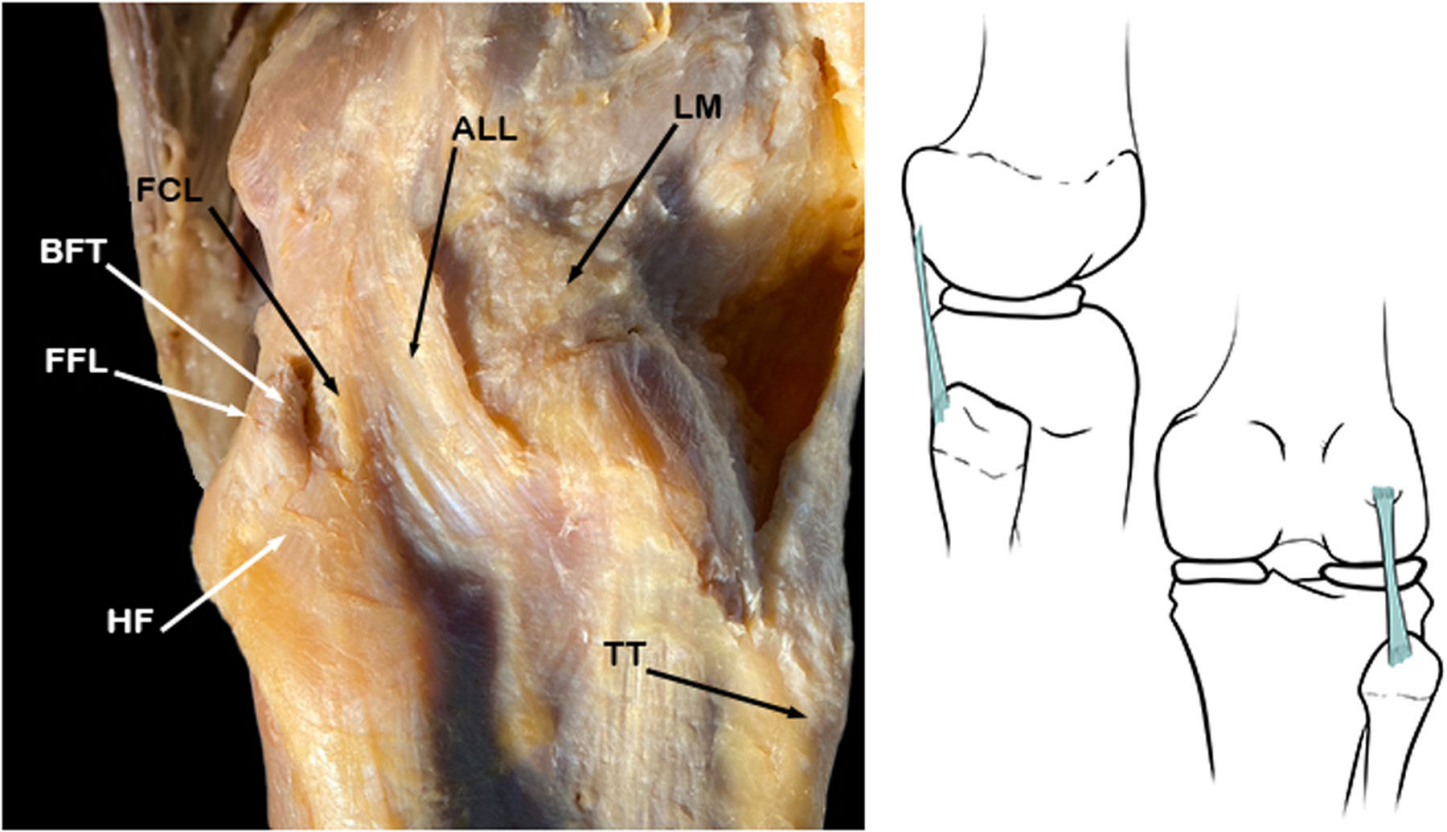
Schematic drawing and anatomical dissection image corresponding to a lateral view of the knee joint to show the Fabellofibular ligament (FFL). ALL, Anterolateral ligament; BFT, biceps femoris tendon; FCL, fibular collateral ligament; HF, head of fibula; LM, lateral meniscus; TT, tibial tuberosity.

﻿The FFL arises from the fabella, an anatomically variable sesamoid bone located predominantly within the head of the origin of the lateral gastrocnemius. It blends with the fibres of the lateral head of the gastrocnemius muscle and runs vertically toward the posterolateral border of the apex of head of fibula [19, 41, 64, 76, 106] (Figure 2).

It was initially believed that this ligament was only found in patients who presented a fabella [7, 17, 64, 85]. However, later works have shown that the presence of the fabella is not a *sine qua non* condition for the presence of this ligament [7, 63, 64, 82, 94, 103, 106]. It has been shown that in cases in which there was no fabella but there was an FFL, it arose from the posterolateral surface of the lateral femoral condyle [7, 103].

The FFL is a component of the PLC that functions as an anatomically variable static stabilizer [73]. Studies have shown no statistically significant relationship between fabella size and FFL thickness. Despite this, hard and bony fabellae are more likely to be accompanied by an FFL than the elastic and cartilaginous fabellae [61].

Several authors have reported the presence of the FFL, but its prevalence in the general population has not yet been determined. Knowing the prevalence rates and anatomical geometry of the FFL is said to help surgeons repair PLC injuries [73].

Due to the contemporary consensus that the fabella is not a requirement for the presence of the FFL, some authors have suggested the name ‘short lateral ligament’ for this structure [42, 53, 83, 85]. There are even studies that define the FFL as a thickening of the capsular arm of the biceps femoris muscle in its distal course towards the fibula. However, the most frequently used name for this structure is FFL due to its attachments on to the fabella and fibula [40, 42]. This is also the terminology for the ligament listed in TA2 [22].

Recent studies have proposed a new nomenclature for this ligament, the *gastrocnemius‐fibular ligament*. They argue that it is more appropriate because of the consistent attachment of the ligament at the apex of the head of the fibula and the head of the lateral gastrocnemius, regardless of the presence or absence of the fabella [73].

#### Anterolateral ligament

In 1879, Segond, a French surgeon, described the presence of a ‘tough fibrous band’ on the anterolateral aspect of the human knee. It inserted on the anterolateral aspect of the proximal tibia, the avulsion of which is known with the eponymous of Segond's fracture. Segond's fracture is defined as a bony avulsion of the tibial attachment of the ALL [15].

**Figure 3:**
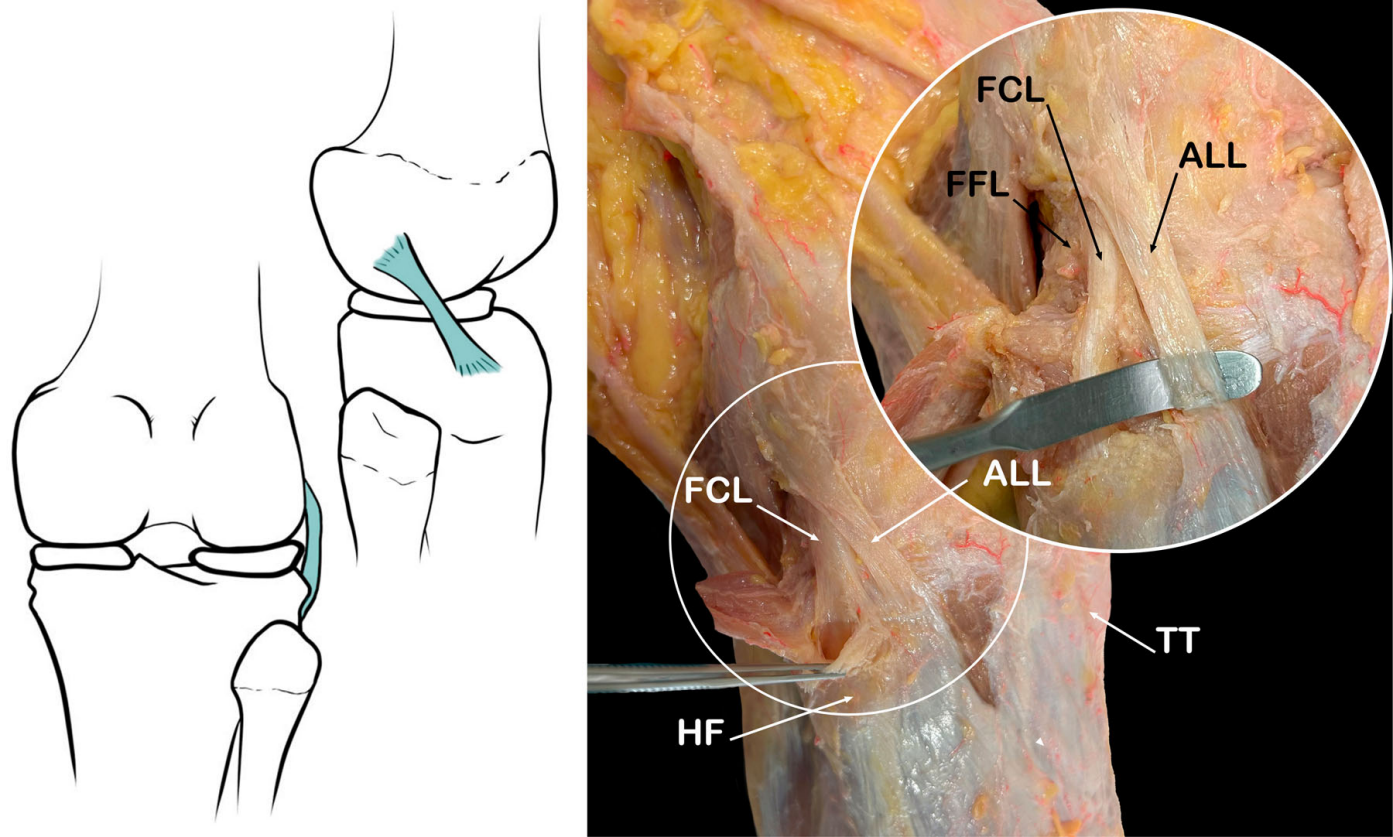
Schematic drawing and anatomical dissection image showing the Anterolateral ligament (AAL). FCL, fibular collateral ligament; FFL, Fabellofibular ligament; HF, head of fibula; TT, tibial tuberosity.

The ALL is a well‐defined fibrous band distinguishable from the anterolateral capsule even though many articles define it as a thickening of the lateral capsule [3, 13]. Anatomic studies have yielded conflicting results in terms of prevalence, with it ranging from 0% to 100% [71]. In our series, the ALL was present in almost 70% of the knees. It is part of the lateral capsule as it thickens and extends along the femur, with its femoral attachment located just anterior to the insertion of the PT onto the lateral epicondyle [47, 94]. The femoral attachment of this ligament occurs precisely at the prominence of the lateral epicondyle of the femur, slightly anterior to the attachment of the FCL. Some connecting fibres between both structures are observed. It extends distally with an oblique trajectory, with strong attachments to the external meniscus. It eventually reaches the anterolateral aspect of the tibia slightly posterior to Gerdy's tubercle. This attachment point is localized between Gerdy's tubercle and the apex of the fibular head [15]. In addition, various authors describe it as being inserted on the lateral meniscus with two types of fibres: a meniscofemoral component and a meniscotibial component [15, 28, 47, 62, 79, 94]. Some authors consider the lateral tibial attachment of the external meniscus to be provided by the meniscotibial portion of the ALL [83, 94]. LaPrade distinguished this structure from the coronary ligament by stating that the coronary ligament is located posterior to the ALL [83, 103]. The ALL has also been identified in MRI studies (Figure 3).

The biomechanics of this ligament are still not well‐established. However, it is hypothesized that ALL controls the medial rotation of the tibia and acts as a secondary stabilizer of the knee [15, 48]. The mechanical and microstructural properties of the ALL seem to be more like those of the anterolateral capsule rather than the properties of the FCL [71]. Recent studies have demonstrated a rich innervation of the ALL which suggests an active role of the ligament in the knee's proprioception [5]. In this way, to repair the ALL in cases of Anterior cruciate ligament rupture reduces post‐surgical instability. It has also been related to a lower rate of plastia failure.

A discrepancy in the terminology used to refer to this structure has been observed. Throughout the reviewed literature, this structure has been given distinct names. They include the mid‐third lateral capsular ligament, lateral bony‐capsular layer of the iliotibial tract (ITT), lateral capsular ligament, or ALL, as used in this manuscript [15].

#### Arcuate popliteal ligament

**Figure 4:**
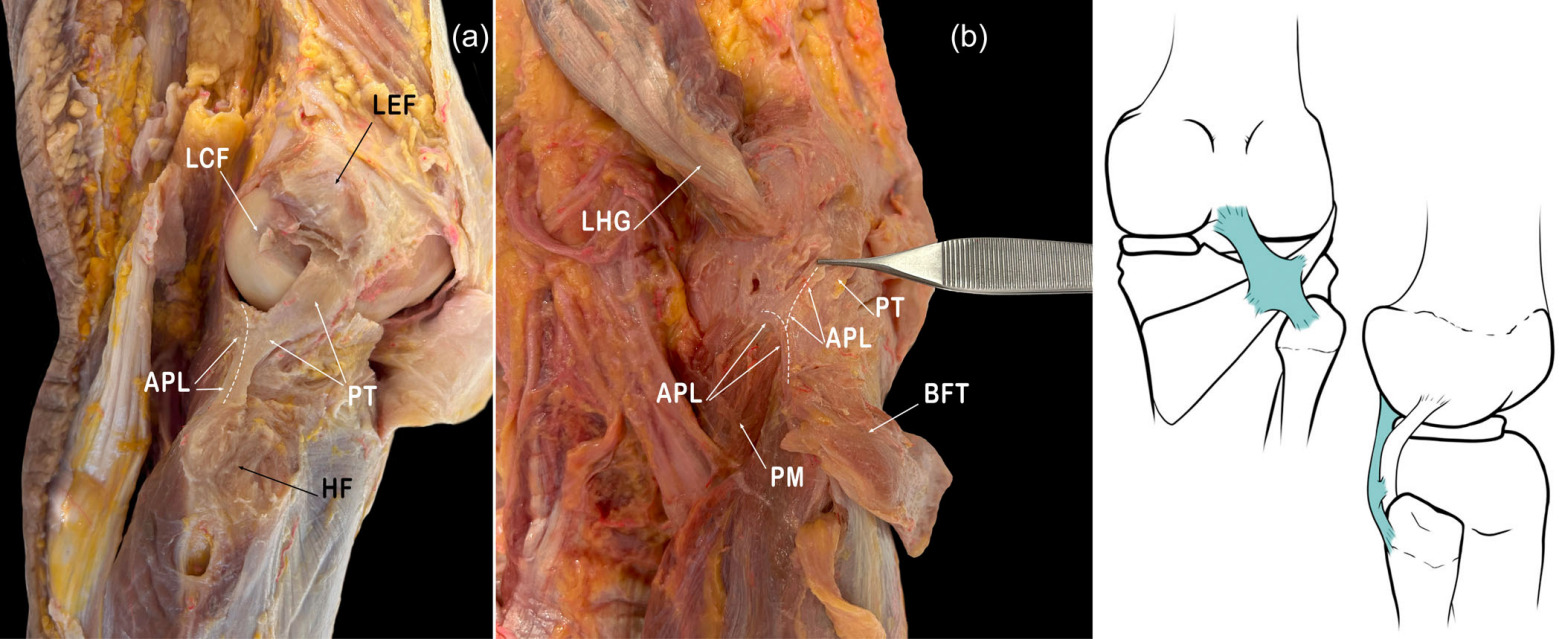
Anatomical images and scheme showing the disposition of the Arcuate popliteal ligament (APL). HF, head of fibula; LCF, lateral condyle of femur; LEF, lateral epicondyle of femur; LHG, lateral head of gastrocnemius; PM, popliteus muscle; PT, popliteus tendon.

In 1948, Last defined the APL as a condensation of the posterolateral aspect of the articular capsule of the knee with a variable thickness and often in a Y‐shaped structure. The distal attachment of this ligament is in the apex of the head of the fibula, just posterior to the FCL. Some fibres blend with the origin of the lateral head of gastrocnemius, whereas other fibres fuse with the posterolateral capsule in the region of the lateral femoral condyle. A component of the ligament arches over the popliteus muscle, where it lies on it and firmly attaches to the popliteus fascia. The popliteus muscle gives rise to some fibres of this ligament. Most of the lateral fibres are thin and occasionally separate from the ligament itself. Some authors have named these fibres the short lateral collateral ligament when they occur. The inferior lateral genicular vessels pass superficially to this short lateral collateral ligament but deep to the FCL. The APL is firmly attached to its upper end at the posterior arch of the lateral meniscus [53] (Figure 4).

The Federative International Programme for Anatomical Terminology (FIPAT) refers to this ligament as the APL [22]. However, reports in the literature have also denominated it variously as the arcuate ligament, the short external lateral ligament, the deep part of the lateral ligament, or the popliteal ligament [53, 96]. Some authors have employed the term ‘Arcuate ligament complex’. But it is necessary to know that this concept refers to an anatomical entity consisting of the PFL, the FFL, the ALL, the Popliteomeniscal fascicles, and the Lateral meniscotibial fibres. It is necessary to note that when some authors talk about the Arcuate ligament, they are really talking about the entire Arcuate ligament complex [96].

#### Popliteofibular ligament

Higgins first described the fibular attachment of the Popliteus muscle tendon in 1984 [35]. The literature surrounding this structure is full of descriptions, omissions, confusion, and rediscoveries. In 1950, Last et al. published a description of the PFL, which they named the *Short external lateral ligament* [54]. However, the International Congress of Anatomists celebrated in London in 1955 omitted the existence of this “Short external lateral ligament”. Recent consensus around the PLC considered the PFL one of its three primary structures [52].

**Figure 5:**
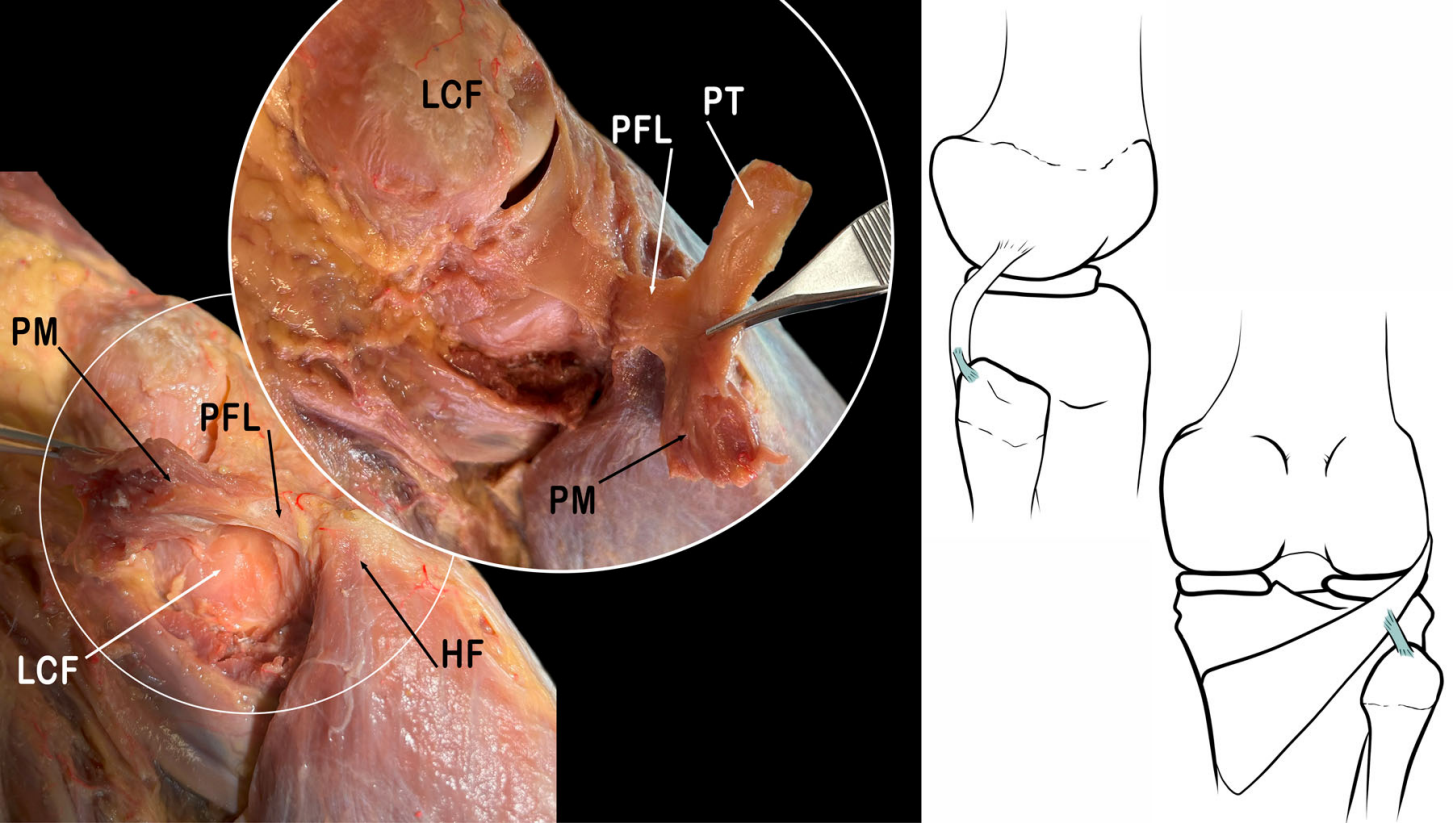
Anatomical image and schematic drawing showing the Popliteofibular ligament (PFL). HF, head of fibula; LCF, lateral condyle of femur; PM, popliteus muscle; PT, popliteus tendon.

The PFL arises from the popliteus muscle tendon at the Musculotendinous junction and inserts on the posterior and medial aspect of the apex of the head of the fibula, posterior to the FCL attachment [48]. It is most frequently described as consisting of two fascicles, anterior and posterior. Functionally, it acts as a stabilizer of the knee during lateral rotation [37, 64]. Stäubli and Birrer reported that the posterior branch of the PFL attaches to the anterior popliteomeniscal fascicle (A‐PMF) proximally, thus describing a short connection between the meniscus and the fibular head [91] (Figure 5).

Based on cadaveric studies, the ligament has been described as a strong structure with an average length of 10–14 mm with an average anteroposterior diameter of 7–9 mm and an average thickness of about 2 mm [75]. The PFL has been identified in almost 100% of dissected knees [19, 37, 64]. However, of the structures that make up the PLC, the PFL has given rise the most confusion and controversy [48].

The PFL has been described as having at least three morphologies. They are the Inverted Y‐shaped ligament, the PFL consisting of a double layer, and a single‐layer PFL. A better anatomical understanding of this ligament, its prevalence, and its morphological diversity would improve the prognosis, diagnosis and treatment of PLC injuries [73].

The name we should use to refer to this structure is PFL, according to the FIPAT [22]. However, the revision of the literature shows it has been named variously the Short external lateral ligament [63, 89], Popliteofibular fascicle [63, 91], and Popliteofibular muscle arising from the fibula [63, 103] or Popliteofibular fibres. On the other hand, the name Short external lateral ligament had been employed to refer to the PFL [63], the FFL [42], and, occasionally, the term arcuate ligament had been used to describe the posterior fibres of this short external lateral ligament in older literature. This highlights the confusion around the PFL even today.

#### Meniscofibular ligament

Zivanovic was the first to describe the MFL in 1964 and determined an incidence rate of 78% in 241 knees he had examined. On the other hand, the MFL has been found in all specimens in other cadaveric studies. The differences in the prevalence of this ligament in classical textbooks and other studies might be explained by the fact that the MFL is a thin and almost undetectable ligament in some specimens. The surgical microscope allows one to visualize the MFL despite its thinness [8, 67, 68].

The MFL is a trapezoidal capsular ligament anterior to the PT. It arises in the inferolateral border of the external meniscus and inserts on the head of the fibula. This attachment is deeper than the distal attachment of the FCL. The MFL belongs to the deep layer of structures that make up the PLC of the knee [24, 68, 69, 98, 107].

Regarding the morphological characteristics, Zivanovic (quoted by Natsis et al. [68]) found that the average width was 8–13 mm, and the average length was 13–22 mm. The author also stated that the size of the MFL depended on the age and height of the individual examined without providing further data [67]. Recent studies state that the average thickness of the MFL, including the capsule to which it is attached, is 3.84 mm [69].

**Figure 6:**
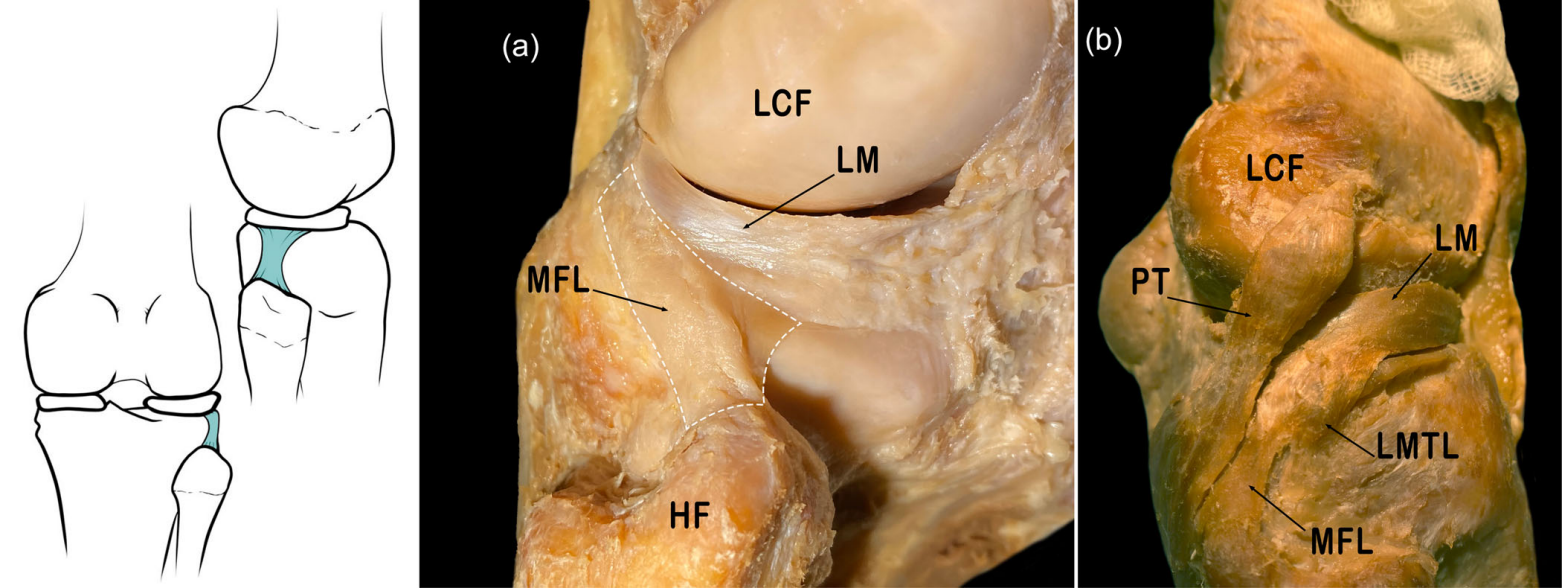
Schematic drawing and anatomical dissection images of the posterolateral corner where the meniscofibular ligament (MFL) can be observed. HF, head of fibula; LCF, lateral condyle of femur; LM, lateral meniscus; LMTL, lateral meniscotibial ligament; PT, popliteus tendon.

From a histological point of view, it consists of dense, regular connective tissue with little extracellular matrix and few elastic fibres in the lower part of the ligament [67] (Figure 6).

Some authors have discussed the influence of the proximal tibiofibular joint on the presence and characteristics of the MFL [8, 67, 68, 92, 98]. In those knees with an oblique proximal tibiofibular joint, the MFL was thinner, less than 3.7 mm on average, than in those knees with a horizontal proximal tibiofibular joint. The latter MFL was thicker, greater than 3.7 mm on average [8, 98]. These differences could be related to the difference in tension that the MFL ligament experiences because of the difference in the position of the fibula [8].

Bozkurt et al. stated that the MFL might be responsible for posterior and lateral displacement of the lateral meniscus as the fibula rotates laterally during dorsiflexion of the ankle joint. The MFL seems to have a protective effect in varus and lateral rotation trauma. However, it may cause repetitive lateral meniscus tears [8]. The MFL may protect the lateral meniscus from damage during the later phases of knee extension [67]. When the knee is flexed, the posterior margin of the MFL is positioned more horizontally, whereas knee extension results in a more vertical and taut position of the ligament [107].

The MFL and the lateral meniscotibial ligament (LMTL) converge at their proximal attachment and split into distinct structures distally [107]. The MFL reinforces the posterolateral part of the LMTL, which may explain the relatively low incidence of MFL tears [67]. Some studies suggest that the MFL is involved in either the presence or absence of lateral meniscus tears [8]. However, no consensus has been established on the precise function of the MFL.

The MFL is not listed in the FIPAT anatomical terminology [22], which increases the discrepancy regarding its nomenclature. Throughout the literature, we have found that it is sometimes referred to as a Meniscofibular fascicle rather than a ‘ligament’. That might be due to its little‐studied structural characteristics, ambiguous thickness, the inconsistent terminology surrounding it, and the macroscopic appearance that is closer to the Popliteomeniscal fascicle than to other capsular ligaments [24].

#### Meniscotibial ligaments

**Figure 7:**
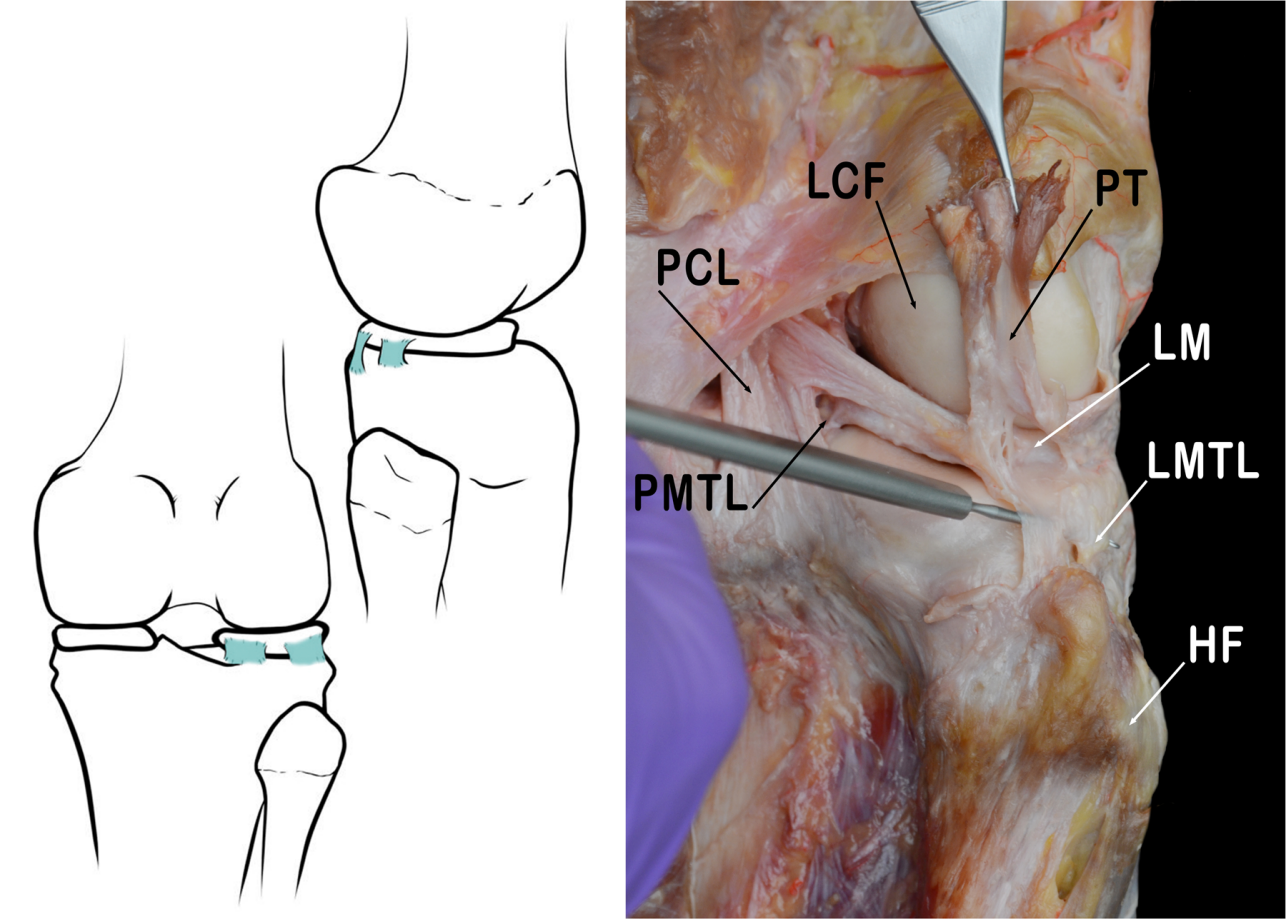
Schematic drawing and anatomical view of the posterolateral corner showing the posterior and lateral menicotibial ligaments (PMTL, LMTL). HF, head of fibula; LCF, lateral condyle of femur; LM, lateral meniscus; PCL, posterior cruciate ligament; PT, popliteal tendon.

The MTL are short connective fibres that attach to the meniscus body peripherally and stabilize the meniscus properly on the tibial plateau [50, 65]. They make up part of the deepest layer of the lateral joint capsule [50, 60, 84]. The fibres of the MTL are challenging to separate from the adjacent fibres of the capsule and the FCL. These ligaments are attached to the tibia a few millimetres below the articular cartilage and sometimes give rise to a small synovial recess [6, 21, 55] (Figure 7).

FIPAT refers to these ligaments as the ‘Lateral meniscotibial ligament’ and the ‘Medial meniscotibial ligament’. Then again, the terms lateral and medial coronary ligament are also accepted [22]. The literature also mentions a third meniscotibial ligament, not referred to by the FIPAT, known as the posterior meniscotibial ligament. This ligament and the lateral MTL represent the posterior and anterolateral attachments of the inferior margin of the external meniscus on the tibial plateau [24].

− The Posterior meniscotibial ligament (PMTL) connects the most posterior and medial segment of the inferior border of the lateral meniscus to the tibial plateau near the attachment of the posterior cruciate ligament. Thus, it provides stability to the posterior meniscal segment. It continues laterally along with the posteroinferior Popliteo‐meniscal fascicle to give continuity to the circumferential restraints of the meniscus.
− The Lateral meniscotibial ligament (LMTL) joins the inferior meniscal surface of the lateral meniscus to the tibial plateau. It starts nearly at the midpoint of the meniscal length and runs toward its anterior portion. Some authors call this the so‐called Lateral coronary ligament. Then again, the acronym LMTL has been identified by some authors as more appropriate to describe its anatomical characteristics.

#### The Menisco‐tibio‐popliteus‐fibular complex (MTPFC)

Recently, a description of the MTPFC was published. It consists of an anatomical complex that is made up of the LMTL, the PFL and the popliteomeniscal fascicles (which the authors call the popliteomeniscal ligament). Greater understanding of the anatomy of this complex and the treatment of its injuries could prevent meniscal extrusion after PLC injuries [58].

#### Meniscofemoral ligaments

The lateral meniscus is connected to the femur through two ligaments of variable strength. They are the MFmL [4, 26]. They are given that name based on their relationship with the posterior cruciate ligament: the anterior (Humphrey's ligament) and the posterior MFmL (Wrisberg's ligament) [20].

The anterior MFmL usually arises from the medial femoral condyle, but an attachment on the Posterior cruciate ligament is also common [2, 10]. It inserts at the posterior horn of the lateral meniscus [23]. The posterior MFmL arises directly proximally to the medial intercondylar ridge at the proximal portion of the posteromedial bundle of the posterior cruciate ligament [2]. Its attachment also occurs at the posterior horn of the lateral meniscus. It has been reported that the anterior MFmL is 21.6–28.3 mm long and the posterior MFmL has a length of 23.4–31.2 mm [18].

There needs to be more consensus in the literature about the prevalence of these two ligaments in humans, either separately or together. Heller [34] found that at least one MFmL was present in 71% of their specimens. The prevalence of the MFmL in other series ranges from 64% [77] to 82% [55]. Some authors even state that the presence of at least one of the MFmLs has an incidence of 100% [44, 102].

The function of the MFmL is not well known [20, 23, 30, 99], and their clinical significance in knee biomechanics is still under study. Some anatomical and biomechanical studies have revealed the importance of MFmL in supporting knee stability. It is hypothesized that they play an important role in protecting the lateral meniscus and increasing the function of the Posterior cruciate ligament [18]. MFmL are considered stabilizers of the posterior horn of the lateral meniscus [55]. In this sense, the MTL and the popliteomeniscal ligament have received particular attention recently [88, 89, 91, 94]. These meniscal attachments may help keep the lateral meniscus in place, thus preventing its extrusion during flexion–extension. Although the ultimate consequences of meniscal extrusion are poorly understood, every effort should be made to avoid this radial displacement [58].

### Myotendinous structures of the PLC

The myotendinous structures present in the PLC are the tendon of the biceps femoris muscle, the ITT, the lateral head of gastrocnemius muscle and the so‐called ‘popliteus muscle complex’. The latter consists of the popliteus muscle tendon with its fascicles (anterior, posterosuperior and posteroinferior) together with the PFL [12, 24, 39].

#### Biceps femoris tendon

﻿It is well known that the biceps femoris muscle is a strong flexor of the knee joint with two heads of origin, long and short. The actions of this muscle are hip extension, lateral rotation of the leg, and it has been shown to play an increased role in anterior cruciate ligament‐deficient knees. ﻿The long head shares its origin with the semitendinosus muscle from the ischial tuberosity and the inferior aspect of the Sacrotuberous ligament. It crosses behind the sciatic nerve from the medial to the lateral side, and fuses with the short head that arises from the lateral lip of the *linea aspera* and from the lateral intermuscular septum [57, 90].

There is no common consensus about the insertion of the Biceps femoris tendon. Most anatomy textbooks put the insertion site of the biceps femoris on the head of the fibula. Furthermore, some authors also describe an additional insertion on the proximal tibia. The long and short heads of the biceps femoris have different insertion points along their course through the posterolateral aspect of the knee [97].

A. The long head of the biceps femoris has three important anatomical attachments in the PLC:

− The direct insertion is at the apex of the head of the fibula.
− The anterior arm, which inserts lateral to the FCL
− The aponeurotic arm, which inserts into the postero‐lateral aspect of the FCL.

**Figure 8:**
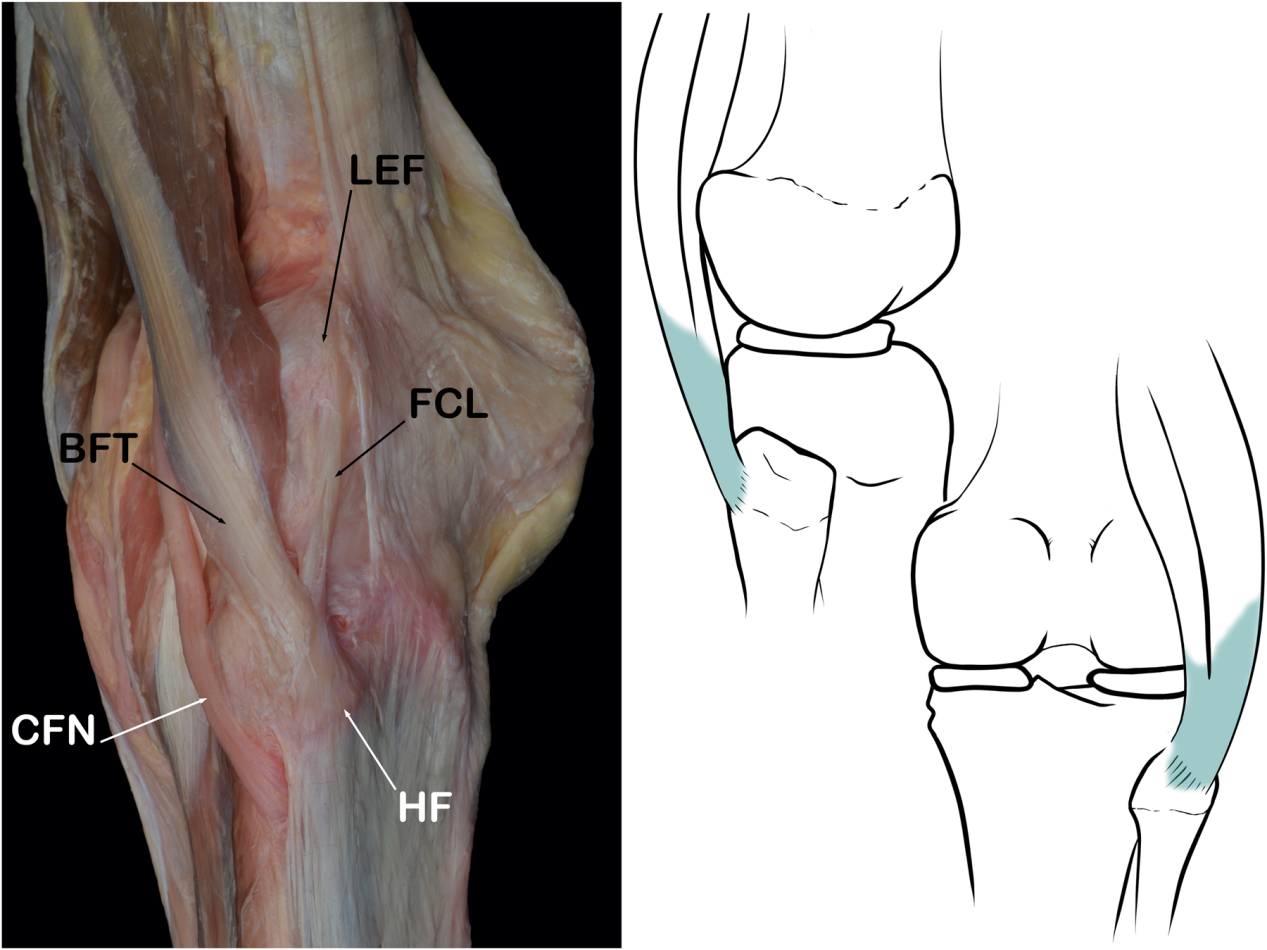
Schematic drawing and dissection image showing the biceps femoris tendon (BFT). CFN, common fibular nerve; FCL, fibular collateral ligament; HF, head of fibula; LEF, lateral epicondyle of femur.

B. The Short head of the biceps femoris also has three major insertions into the PLC of the knee:

− The capsular arm is anchored to the posterolateral capsule, lateral to the apex of head of the fibula. It provides a strong attachment to the capsule, the lateral gastrocnemius tendon, and the capsular layer of the ITT [47, 95].
− Its direct insertion is on the lateral aspect of the apex of the fibular head.
− Laprade et al. described the anterior arm that passes medial to the FCL, inserts on the posterolateral aspect of the tibial condyle, and is approximately 1 cm posterior to Gerdy's tubercle [47]. This anterior arm inserts into the tibia at the same point as the ALL. This reference point is usually damaged in Segond's fractures (Figure 8).

Injuries to the biceps femoris muscle have been described in patients with anterolateral and anteromedial knee rotation instability, an instability that frequently appears in PLC injuries [33, 46, 48, 62, 104].

#### Iliotibial tract

The ITT is the most superficial layer of the lateral aspect of the knee. It is a broad band that is attached to Gerdy's tubercle on the anterolateral aspect of the tibia. Different attachments of the ITT like the patella, the lateral intermuscular septum, and the capsule have been described [13].

**Figure 9:**
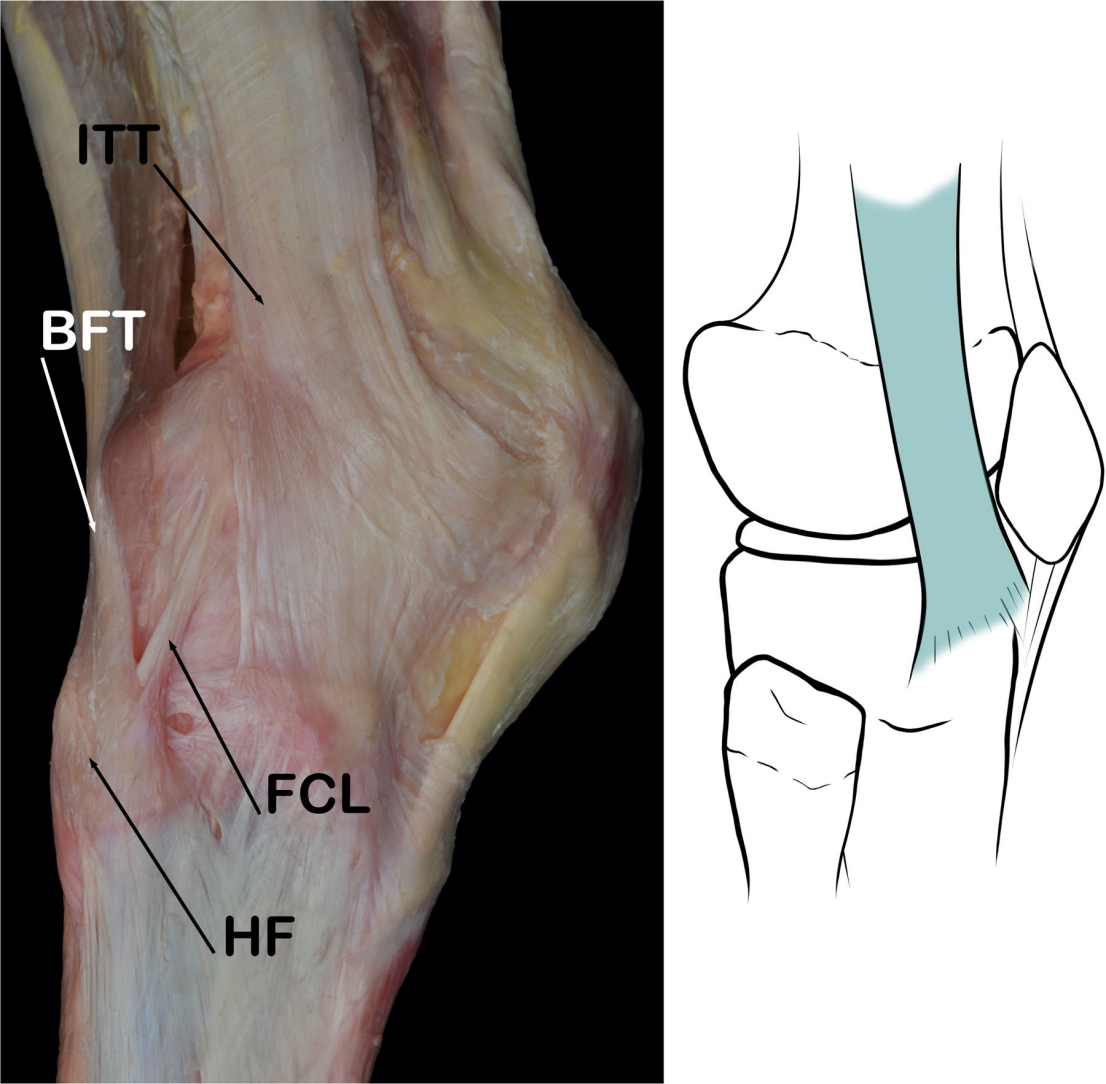
Anatomical image and schematic draw showing the iliotibial tract (ITT). BFT, biceps femoris tendon; FCL, fibular collateral ligament; HF, head of fibula.

The ITT is mainly divided into two layers, the superficial and the capsular. The superficial layer runs along the lateral aspect of the knee attaches to Gerdy's tubercle and extends deeper and attaches to the Lateral intermuscular septum. The capsular layer extends from the intermuscular septum and merges with the short head of the biceps femoris, thus joining it at the anterolateral aspect of the tibia. The ITT stabilizes the posterolateral corner, helping to prevent varus opening (Figure 9).

FIPAT states that it should be referred to as the Iliotibial band or ITT [22].

#### Tendon of the popliteus muscle

**Figure 10:**
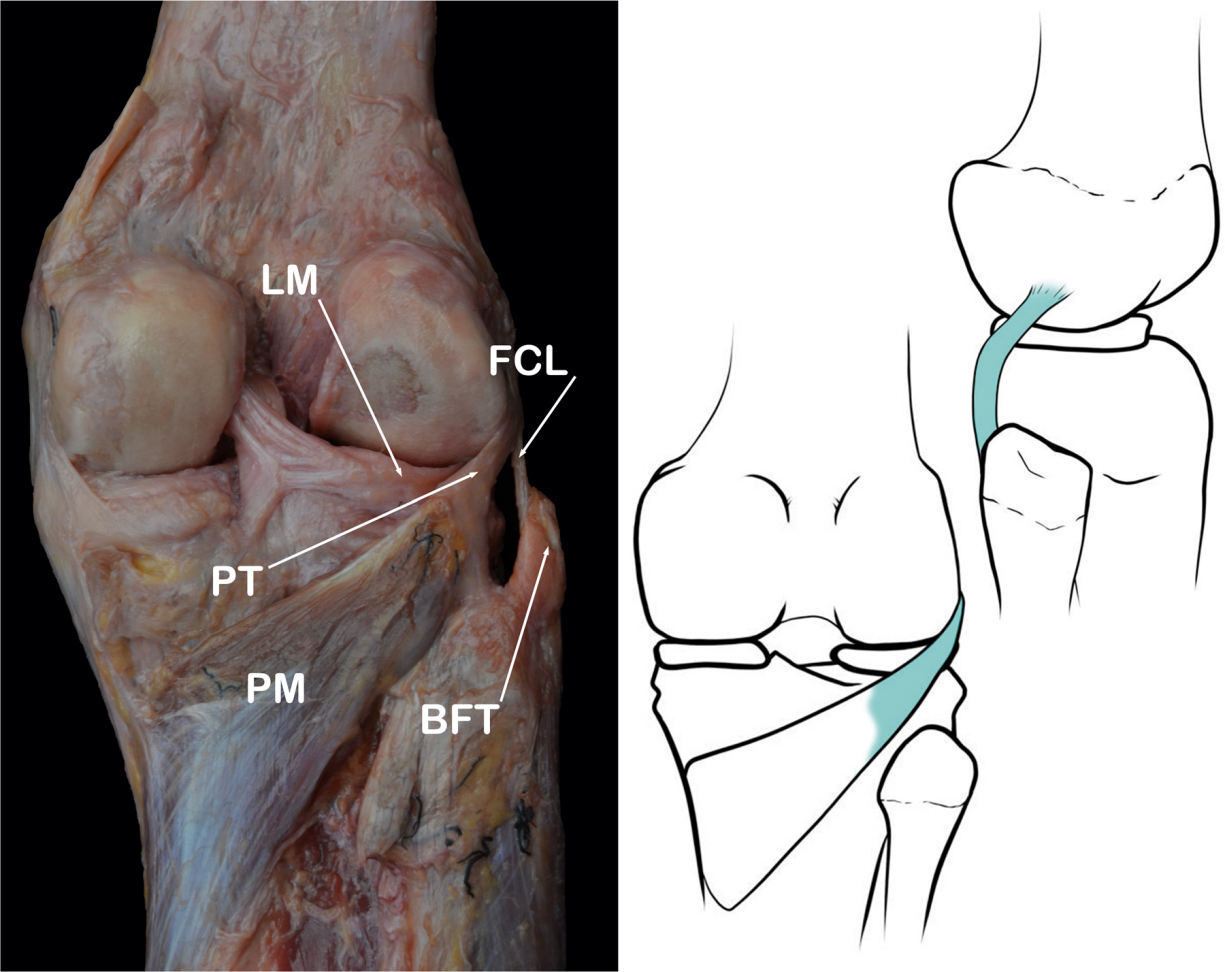
Schematic drawing and anatomical dissection of the popliteus muscle tendon. BFT, biceps femoris tendon; FCL, fibular collateral ligament; LM, lateral meniscus; PM, popliteal muscle; PT, popliteal tendon.

﻿The popliteus muscle is a thin, flat, triangular muscle that is part of the deep layer of the popliteal space. The popliteus muscle has a wide attachment on the surface of the posteromedial tibial, proximal to the soleal line. It forms the floor of the popliteal fossa. It continues superiorly and laterally, to form a long and strong tendon that enters the knee through the popliteal hiatus under the APL. ﻿The PT passes beneath the FCL and the tendon of the biceps femoris muscle. It inserts in a depression on the outer side of the lateral ﻿condyle of the femur [38]. The femoral insertion of the PT represents the most anterior femoral insertion of the PLC structures. The work of LaPrade et al. [48] clearly determined the exact point of insertion of the PT, which is 18.5 mm anterior (17–23 mm) to the attachment of the FCL with the knee at 70° of flexion. After its femoral insertion of origin in the proximal half of the popliteal sulcus, it runs posteriorly and obliquely to insert into the posteromedial surface of the tibia. The myotendinous junction coincides with the passage of the PT muscle under the FCL and the APL, where it becomes muscle. The average total length of the PT to its musculotendinous junction is 54.5 mm [48] (Figure 10).

The PT is a static and dynamic stabilizer of the knee during posterolateral rotation. Some authors state that as the tendon runs posteriorly and distally it may give rise to three fascicles that attach to and stabilize the lateral meniscus [31, 94]. They are called the Popliteomeniscal fascicles, the anterior, postero‐superior, and postero‐inferior [81]. Single insertions of the PT in the lateral meniscus had been already described by Innes in 1818 [36].

#### Anterior popliteomeniscal fascicle

**Figure 11:**
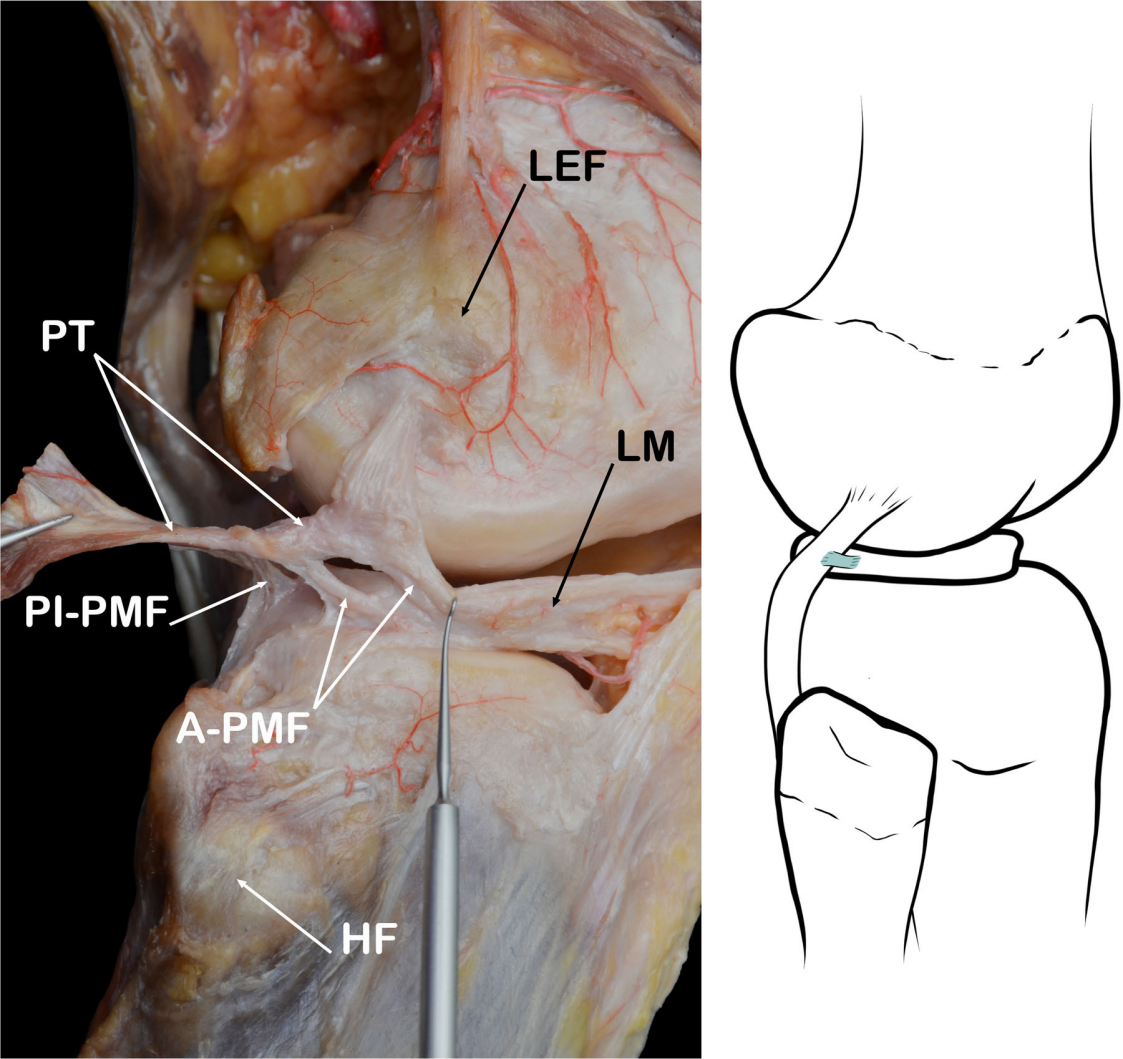
Images showing the anterior‐popliteomeniscal fascicle (A‐PMF). In the anatomical dissection the fascicle was divided into two bands. HF, head of fibula; LEF, lateral epicondyle of femur; LM, lateral meniscus; PI‐PMF, postero inferior popliteomeniscal fascicle; PT, popliteus tendon.

This fascicle originates from the inferomedial portion of the PT and inserts on the external surface of the lateral meniscus, thus delimiting the anterior margin of the superior opening of the popliteal hiatus. The course of its meniscal insertion is oblique, with a posteroinferior to anterosuperior direction, and it has been reported it has an average length of 8.0 mm ± 1.9 mm [1]. This oblique course of the PT is the one it takes when it becomes intra‐articular. Inferiorly, the A‐PMF blends with the meniscofibular fascicle to create a junction that reaches the fibular head (Figure 11).

#### Postero‐superior popliteomeniscal fascicle (PS‐PMF)

**Figure 12:**
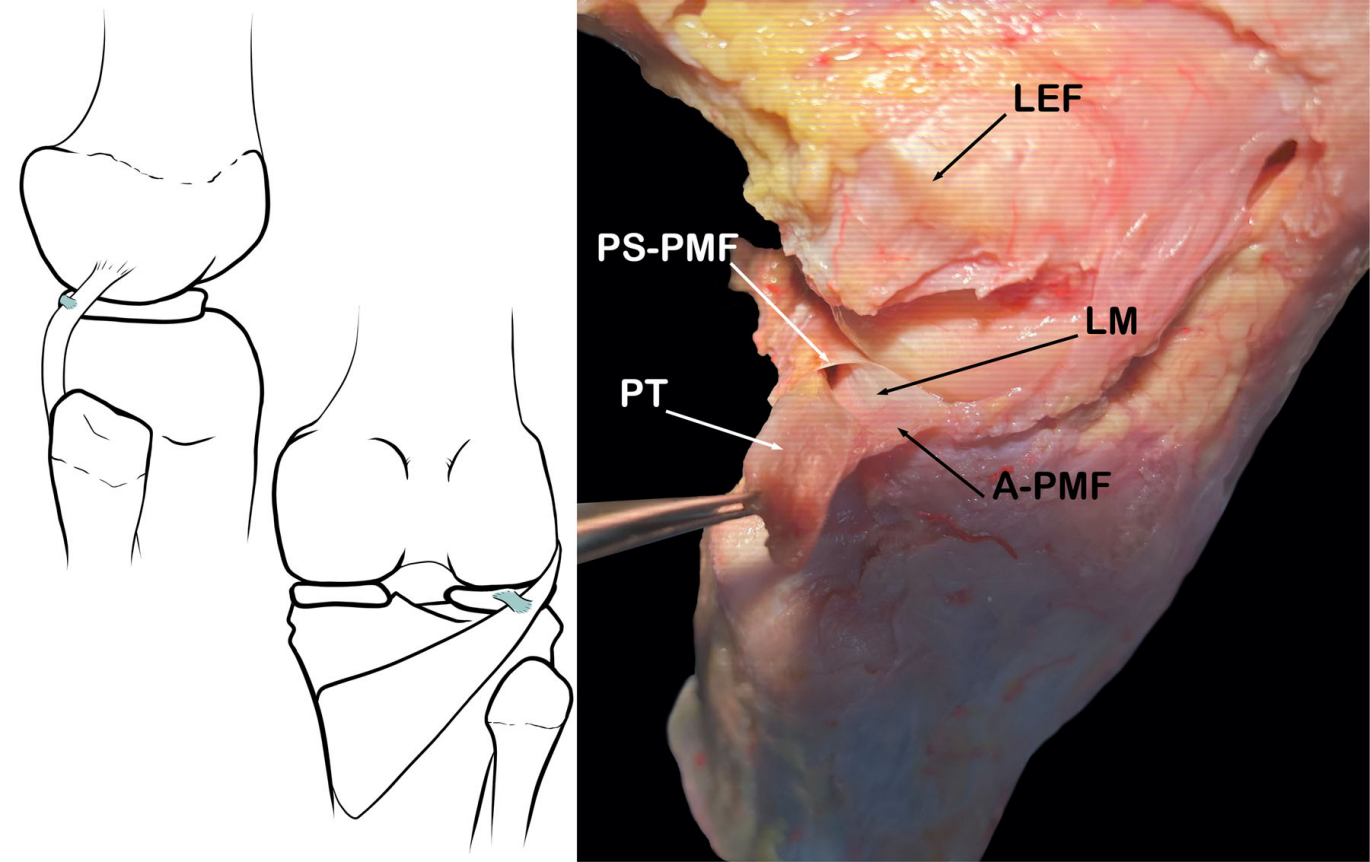
Schematic drawing and anatomical dissection showing the postero‐superior popliteomeniscal fascicle (PS‐PMF). A‐PMF, anterior popliteomeniscal fascicle; LEF, lateral epicondyle of femur; LM, lateral meniscus; PT, popliteus tendon.

This fascicle originates on the posterior surface of the PT and ends mainly at the top of the posterior horn of the meniscus. In addition, it covers the most medial part of the PT. The average length of its attachment to the lateral meniscus is 6.5 ± 1.5 mm [1]. This fascicle, together with the postero‐inferior popliteomeniscal fascicle, creates a small recess behind the external meniscus (Figure 12).

#### Postero‐inferior popliteomeniscal fascicle (PI‐PMF)

This fascicle is believed to be the structure described more than 70 years ago as the ‘quadrilateral aponeurosis’. It connects the inferior margin of the lateral meniscus to the superior and medial portion of the popliteus muscle [53].

According to Aman et al. [1], the average length of the PI‐PMF attachment to the lateral meniscus is 8.5 ± 1.8 mm.

**Figure 13:**
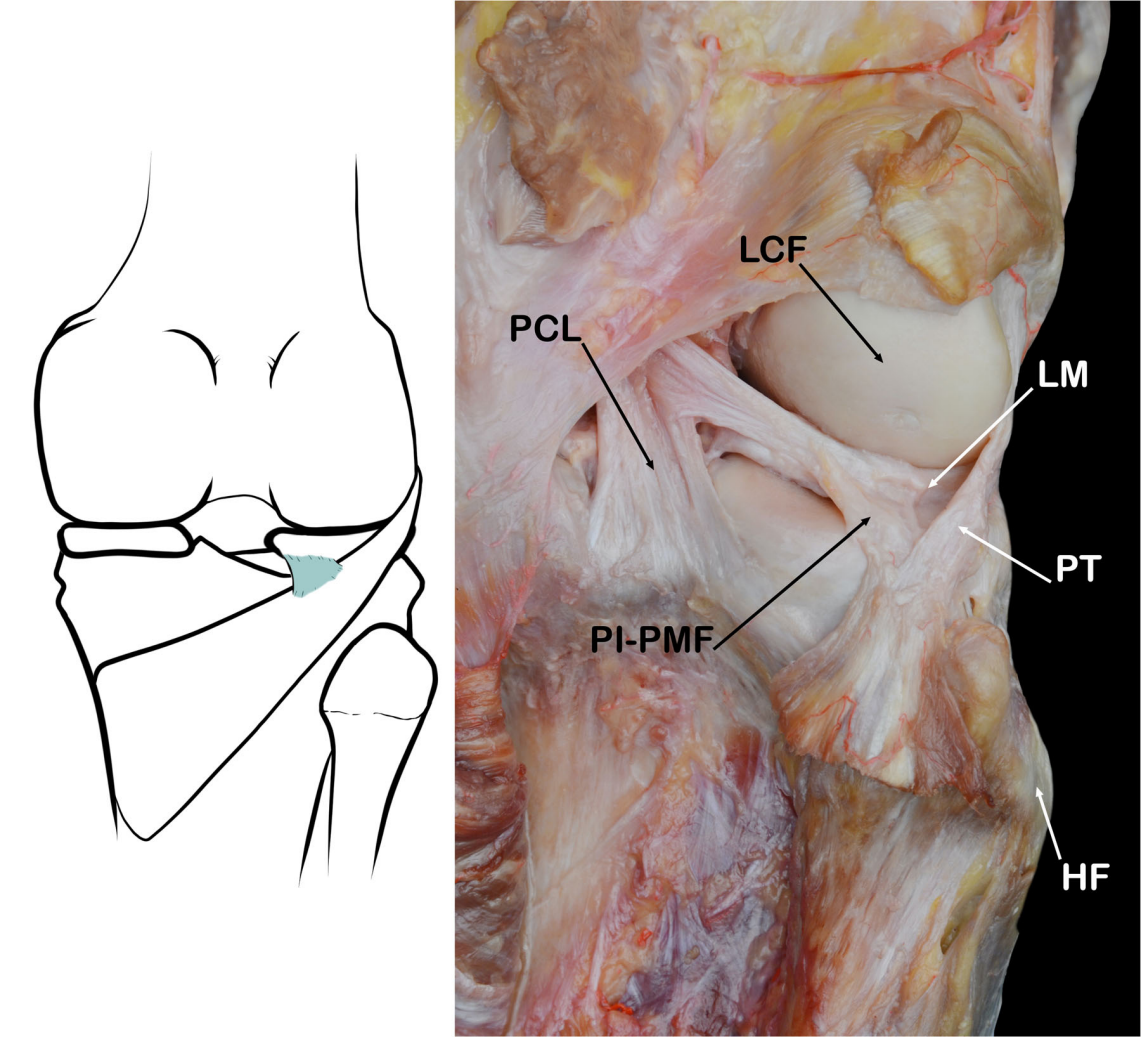
Schematic drawing and anatomical dissection where can be observed the postero‐inferior popliteomeniscal fascicle (PI‐PMF). HF, head of fibula; LM, lateral lemiscus; PCL, posterior cruciate ligament; PT, popliteus tendon.

Inconsistencies in the descriptions of the third popliteomeniscal fascicle and doubts about its existence have been reported [72]. This may be related with a study technique that does not consider the aponeurotic capsular extension medial to the PT. It may also be related to the presence of anatomical variants in which a single complete structure, indistinguishable from the Meniscofibular fascicle, is attached to the inferior border of the posterior meniscus. It would prevent access to the PT below the meniscus from the intra‐articular space [32, 43, 53] (Figure 13).

There are no differences of opinion as to how we should refer to the popliteus muscle, but there are disagreements as to the anatomical descriptions of its fascicles and how the fascicles of the popliteus muscle should be named. Those issues have not been taken up by FIPAT [22]. While there is unanimity throughout the literature in naming the fascicles ‘posterosuperior’ and ‘posteroinferior’, there is a lack of consensus regarding the naming of the most anterior fascicle. It has been called ‘anteroinferior’, ‘anterosuperior’ o simply ‘anterior’ [1, 72, 91].

#### Lateral head of the gastrocnemius muscle

The gastrocnemius muscle has two heads at its origin, the medial and lateral. These heads insert proximally into the posterosuperior region of the corresponding femoral condyle [16]. The lateral head of the gastrocnemius muscle, whose tendon passes through the PLC, originates about 14 mm posterior to the femoral insertion of the FCL along the supracondylar process.

**Figure 14:**
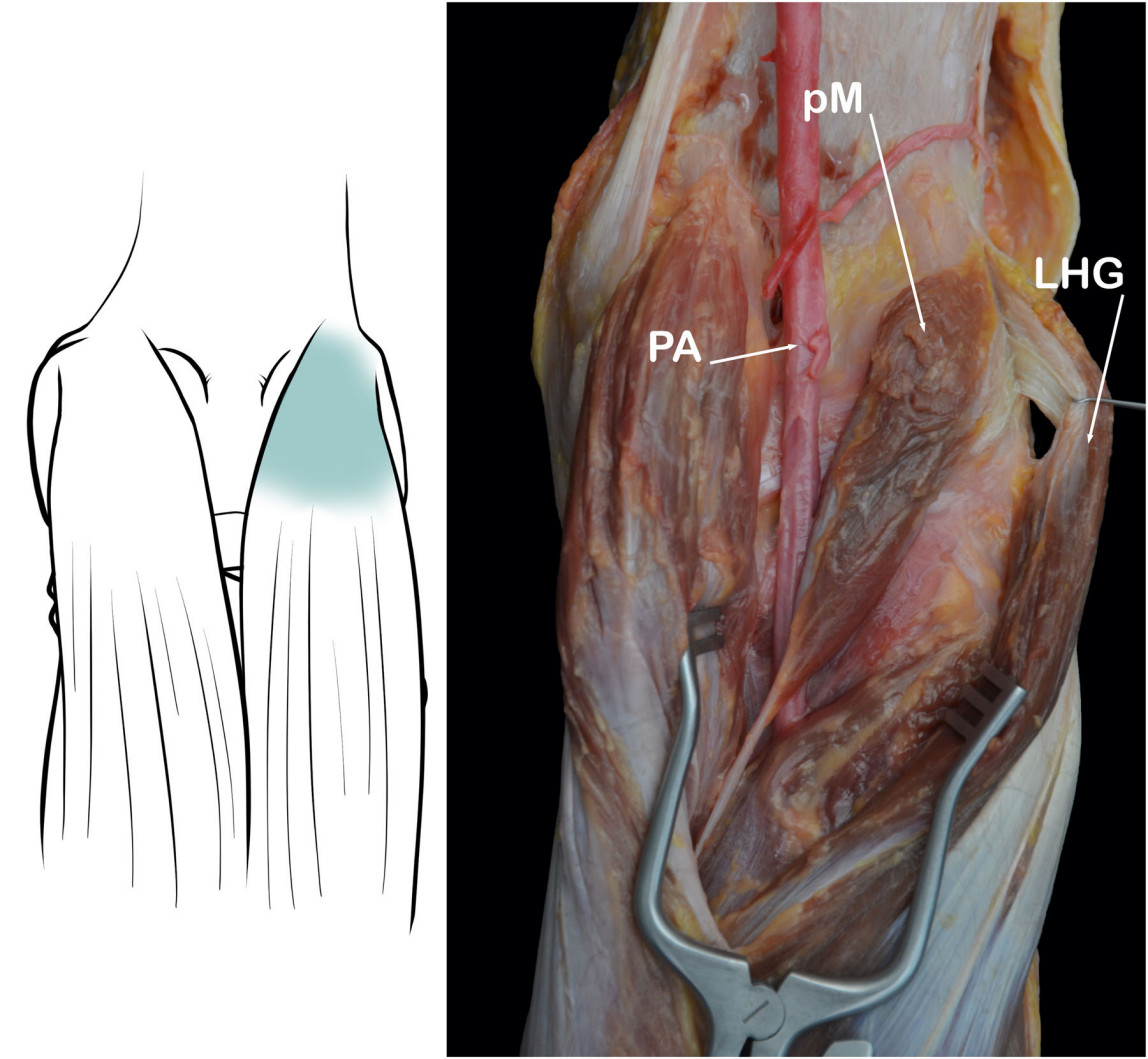
Images showing the anatomy of the lateral head of gastrocnemius (LHG). PA, popliteal artery; pM, plantaris muscle.

The lateral gastrocnemius tendon courses distally (in close relationship with the posterolateral capsule) to fuse with the medial gastrocnemius and soleus to form the triceps suralis (Figure 14). Injuries to this tendon are usually associated with severe trauma and are infrequent. As it is less frequently injured than the other structures of the PLC, it can be an important landmark during surgical reconstruction [13].

There is no discrepancy as to how to name this muscle. The FIPAT lists it in its Anatomical Terminology [22] as the gastrocnemius muscle, and its heads as the lateral and medial heads of the gastrocnemius muscle. This is how we found it throughout the literature.

## DISCUSSION AND CONCLUSIONS

From a clinical point of view, different consensus on PLC injuries have been published. The study of Chahla [14] and, recently, the multicentric work of LaPrade et al. [52] have furnished a consensus on the diagnosis, classification, treatment, and rehabilitation of these kinds of injuries. They consider the FCL, the PT and the PFL the main primary structures in the PLC with a consensus of 100% among the orthopaedic surgeons.

On the contrary, the anatomical descriptions of PLC are still controversial. Not only do the anatomy textbooks describe the different components of the PLC in a different way, but they also make very variable descriptions. On many occasions, only a few structures that belong to the PLC have a clear and consensual definition of their proximal and distal attachments, dimensions, trajectory and so forth. There are also discrepancies regarding the presence and prevalence of these structures in the general population as well as their anatomical variants. In this context, it must be noted that the nomenclature around these structures is not always considered in the current Nomina Anatomica [22].

All these facts have relevant consequences for surgeons and radiologists. The anatomy and biomechanics of the PLC components should be well known to recommend surgical reconstruction in case of injury to the main structures of the PLC. Questions like the junction to external meniscus need to be well described to determine the best surgical strategy. The musculotendineous junctions are also an issue that must be considered when the PLC is surgically repaired [52].

The MRI is the elective technique for the diagnosis of PLC injuries. But this lack of anatomical consensus on the PLC anatomy sometimes makes it difficult to achieve an accurate diagnosis. On the other hand, the variations in terminology are apparent. This only increases and perpetuates the confusion about this anatomical region. Confusion only helps to delay and impede a good prognosis, diagnosis, and subsequent treatment of PLC lesions.

The main limitation of the study is that it is a narrative review without a systematic search strategy. We reviewed the main documents of the last hundred years by doing a bibliographic search using the different terms that have been employed to describe the PLC elements. This has allowed us to show the different ways in which the same anatomical structure has been named in the literature (Table 1). In this sense, the manuscript serves as a useful condensed illustrated report for both anatomists and clinicians.

**Table 1:** Anatomical terms used for PLC structures along the literature.

Anatomical structures in the PLC		Present in TA2	Equivalent used terms along the reviewed literature
Biceps femoris muscle		X	
Iliotibial tract		X	Iliotibial band [22]
Popliteus muscle	Popliteus muscle tendon	X	
	Anterior popliteomeniscal fascicle		
	Postero‐superior popliteomeniscal fascicle		
	Postero‐inferior popliteomeniscal fascicle		Quadrilateral aponeurosis [79]
Lateral head of gastrocnemius muscle		X	
Fibular collateral ligament		X	Lateral collateral ligament [44], fibular lateral ligament [16], fibular collateral ligament [34], fibular collateral lateral ligament [22], degeneration of the long fibularis (peroneus) muscle tendon [45, 46], long lateral ligament of the knee or long external lateral ligament [47].
Fabellofibular ligament		X	Short lateral ligament [47, 54, 62, 63], gastrocnemiofibular ligament [60], thickening of the capsular arm of biceps femoris [22].
Lateral meniscotibial ligament		X	Coronary ligament [30], [119]
Posterior meniscotibial ligament			Coronary ligament [119]
Arcuate popliteal ligament		X	Short external lateral ligament or deep part of the lateral ligament [47] Arcuate ligament [71], popliteal ligament [72] and arcuate complex [40]
Popliteofibular ligament		X	Short external lateral ligament [77], popliteofibular fascicles [75], popliteofibular muscle arising from the fibula [58], or popliteofibular fibres [78]
Meniscofibular ligament			Meniscofibular fascicle [81]
Anterolateral ligament			Mid‐third capsular lateral ligament [22], capsular layer of the tibial band or Anterolateral ligament [65].
Anterior meniscofemoral ligament		X	Humphrey's ligament [97] Roberts' ligament [44]
Posterior meniscofemoral ligament		X	Wrisberg's ligament [97]

## AUTHOR CONTRIBUTIONS

**Xavier Angelats**: Conceptualization, methodology, investigation, data curation, writing—original draft. **Anna Carrera**: Methodology, resources, data curation, review and editing. **R. Shane Tubbs**: Formal analysis, review and editing, supervision. **Joe Iwanaga**: Conceptualization, methodology, supervision. **Marc Franco**: Investigation, data curation. **Keishiro Kikuchi**: Writing—reviewing and editing. **Francisco Reina**: Conceptualization, methodology, writing—review and editing, supervision.

## CONFLICT OF INTEREST STATEMENT

The authors declare no conflicts of interest.

## ETHICS STATEMENT

Please include the name of the institutional review board (IRB) and the approval number. If not applicable, please state so. The authors state that every effort was made to follow all local and international ethical guidelines and laws that pertain to the use of human cadaveric donors in anatomical research. Anatomical images were obtained from bodies of an Official Institutional Body Donation Programme (University of Girona).

## Data Availability

Data sharing not applicable to this article as no datasets were generated or analyzed during the current study.
